# Almond hulls and shells as an alternative fiber source in limit-fed growing beef cattle diets

**DOI:** 10.1093/tas/txae025

**Published:** 2024-02-29

**Authors:** Zachary M Duncan, Zachary L DeBord, Madison G Pflughoeft, Kyler J Suhr, William R Hollenbeck, Frank K Brazle, Haley K Wecker, Chad B Paulk, Evan C Titgemeyer, K C Olson, Dale A Blasi

**Affiliations:** Department of Animal Sciences and Industry, Kansas State University, Manhattan, KS 66506, USA; Department of Animal Sciences and Industry, Kansas State University, Manhattan, KS 66506, USA; Department of Animal Sciences and Industry, Kansas State University, Manhattan, KS 66506, USA; Department of Animal Sciences and Industry, Kansas State University, Manhattan, KS 66506, USA; Department of Animal Sciences and Industry, Kansas State University, Manhattan, KS 66506, USA; Department of Animal Sciences and Industry, Kansas State University, Manhattan, KS 66506, USA; Department of Grain Science and Industry, Kansas State University, Manhattan, KS 66506, USA; Department of Grain Science and Industry, Kansas State University, Manhattan, KS 66506, USA; Department of Animal Sciences and Industry, Kansas State University, Manhattan, KS 66506, USA; Department of Animal Sciences and Industry, Kansas State University, Manhattan, KS 66506, USA; Department of Animal Sciences and Industry, Kansas State University, Manhattan, KS 66506, USA

**Keywords:** almond hulls, growing cattle, limit-feeding

## Abstract

Almond hulls and shells are a by-product of almond production that can be incorporated as a feed ingredient in beef cattle diets. Three experiments were conducted to determine the effects of hammermill screen size on almond hull and shell bulk density and inclusion of ground or non-ground almond hulls and shells in limit-fed growing diets on growth performance, diet digestibility, and ruminal fermentation characteristics of beef cattle. In experiment 1, almond hulls and shells were ground with a laboratory-scale hammermill using no screen, a 11.1-mm screen, a 19.1-mm screen, or a 25.4-mm screen. Each screen-size treatment was ground at three separate time points (*n*= 3 replications/treatment). Grinding almond hulls and shells with no screen increased bulk density by 111% and minimized proportions of fine particles; therefore, almond hulls and shells ground using no screen were included as a treatment in the following experiments. In experiment 2, 364 steers (initial body weight [BW]: 257± 20.7 kg) were blocked by truckload (*n* = 4), stratified by BW, and assigned to pen within block. Pens were randomly assigned to 1 of 4 experimental diets (*n*= 10 pens/treatment). The control diet (CON) contained (DM basis) 39.5% dry-rolled corn, 7.5% supplement, 40% wet-corn gluten feed, and 13% prairie hay. Non-ground (13AH) or ground (13GAH) almond hulls and shells replaced prairie hay and were fed at 13% of diet DM or non-ground almond hulls and shells were fed at 26% of diet DM and replaced 13% prairie hay and 13% dry-rolled corn (26AH). Diets were limit-fed at 2.2% of BW daily (DM basis) for 56 d. Overall average daily gains (ADG) were greater (*P* ≤ 0.05) for CON, 13AH, and 13GAH compared with 26AH. In addition, ADG from days 14 to 56 were greater (*P*= 0.03) for 13GAH and tended to be greater (*P* = 0.09) for 13AH compared with CON. Experiment 3 was a 4 × 4 replicated Latin square in which 8 ruminally cannulated heifers (initial BW = 378 ± 44.0 kg) were fed diets from experiment 2. Apparent dry matter digestibility did not differ (*P* = 0.21) among treatments. Total ruminal volatile fatty acid concentrations were greater (*P* ≤ 0.03) for 13GAH and 13AH compared with 26AH and tended (*P* = 0.06) to be greater for 13GAH compared with CON. Overall, almond hulls and shells can be utilized as an alternative to prairie hay in limit-fed growing diets without negatively influencing rates of gain or diet digestibility.

## Teaser

We evaluated the effects of feeding almond hulls and shells as an alternative to dry-rolled corn or prairie hay in growing beef cattle diets. We determined that almond hulls and shells can be used as an alternative to prairie hay in limit-fed growing diets without negativity influencing growth performance or diet digestibility.

## Introduction

Almonds (*Prunus dulcis*) are a tree nut that grows within a shell surrounded by a hull; nuts, hulls, and shells are separated following harvest (Huang and Lapsley, 2019). California is the world leader in almond production and produced 3.77 billion kg of almond tree fruit during the 2022 harvest year (Huang and Lapsley, 2019; ABC, 2023). Almond nuts, hulls, and shells represent 31%, 49%, and 20% of almond tree fruit weight, respectively; therefore, approximately 1.85 billion kg of almond hulls and 753 million kg of shells were generated in 2022 (ABC, 2023). Almond hulls are marketed based on their crude fiber concentrations. Almond hulls containing ≤ 15% crude fiber (CF; AF basis) are marketed as “prime hulls” whereas almond hulls that contain more than 15% CF but less than 29% CF (AF basis) are marketed as “hulls and shells” (CDFA, 2022).

Currently, almond hulls are marketed to local dairy farms because they contain large concentrations of soluble sugars making them a suitable feed ingredient. Almond shells are less digestible than almond hulls and are commonly used for mulching or animal bedding. Increased environmental regulations and urbanization have reduced the California dairy cattle inventory (MacDonald et al., 2020). A continued reduction in the California dairy cattle herd could decrease almond hull demand; therefore, research evaluating alternatives for almond hulls or almond hulls and shells is warranted.

One possible use for almond hulls and shells is incorporation into beef cattle diets. Growth performance was not negatively impacted when almond hulls replaced portions of alfalfa and oat hay in finishing diets (Beckett et al., 1992); however, research evaluating almond hull and shell inclusion in growing diets is limited. One challenge associated with almond hull and shell inclusion in beef cattle diets is that the bulk density of non-ground almond hulls and shells makes transporting them long distances to cattle-feeding areas difficult and expensive. Grinding almond hulls and shells is a potential solution to increase bulk density so that greater masses of hulls can be transported in a single load. In addition, grinding almond hulls and shells may also improve digestibility, reduce sorting, and improve feed aggregation. Our objective was to evaluate the effects of hammermill screen size on almond hull and shell particle size and bulk density. Additional objectives were to determine the effects of feeding ground or non-ground almond hulls and shells on growth performance, apparent diet digestibility, and ruminal fermentation characteristics in growing calves limit-fed a high-energy diet based on corn and corn co-products.

### Materials and Methods

The Kansas State University Institutional Animal Care and Use Committee reviewed and approved all animal handling and animal care practices used in our experiment. All animal procedures were conducted in accordance with the Guide for the Care and Use of Animals in Agricultural Research and Teaching (FASS, 2020).

### Almond hull composition

The almond hulls used in the following experiments contained between 15.6% and 18.6% CF (AF basis; Table 1) and were considered almond hulls and shells. Almond hulls and shells were obtained from a commercial processer (Parreira Almond Processing Company, Los Banos, CA), included 50% nonpareil and 50% California type varieties, and were grown in Merced and Fresno counties. Almond hulls and shells used in experiments 1 and 3 were received in October 2021, and hulls and shells used in experiment 2 were received in January 2022.

**Table 1.** Composition of almond hulls and shells and prairie hay

	Almond hulls and shells		Prairie hay	
Item, % DM	Experiments 1 and 3	Experiment 2	Experiment 2	Experiment 3
Dry matter	92.1	89.4	90.4	91.6
Crude protein	4.3	4.4	4.6	5.0
Organic matter	93.5	94.7	93.0	93.6
Ash	6.5	5.3	7.0	6.4
Crude fiber	20.2	17.4	-	-
Neutral detergent fiber	33.3	22.6	65.6	61.3
Acid detergent fiber	27.8	17.4	41.4	38.9
Calcium	0.31	0.18	0.51	0.39
Phosphorus	0.05	0.08	0.09	0.09
Potassium	3.36	2.70	0.78	1.34
Magnesium	0.15	0.09	0.14	0.16

#### Experiment 1: almond hull processing

To determine the effects of hammermill-screen size on particle size and bulk density, almond hulls and shells were ground using no screen, a 11.1-mm screen, a 19.1-mm screen, or a 25.4-mm screen using a laboratory scale 1.5 HP Bliss Hammermill (Model 6k630B; Bliss Industries, LLC, Ponca City, OK). Approximately 9 kg of almond hulls and shells were ground for each replication. Each replication was ground at three separate time points to provide three replications per treatment.

Following processing, a subsample of each treatment was analyzed in duplicate for particle size according to ASABE 319.4 methods (ASABE, 2008). A 100 ± 5 g sample and 0.5 g of a flow agent were placed in the top sieve of a 13-sieve stainless steel sieve stack and sifted for ten minutes using a Ro-Tap machine (Model RX-29; W. S. Tyler Industrial Group, Mentor, OH). The sieve stack contained 16-mm rubber balls and bristle sieve cleaners. The proportion of sample remaining in each sieve after sifting was weighed and geometric mean diameter and geometric standard deviation were calculated. In addition, particle size was also evaluated using the Penn State Particle Separator (Heinrichs and Kononoff, 2013). Two hundred g of sample were placed in the top sieve. The box was then shaken five times in one direction, rotated a quarter turn and shaken five more times; this process was repeated seven more times for a total of 40 shakes. Each sieve was weighed to determine the proportion of sample remaining. Lastly, bulk densities of the initial non-ground almond hulls and shells and ground almond hulls and shells were measured as described by Clementson et al. (2010).

#### Experiment 2: growth performance

Three-hundred sixty-four British × continental crossbred steers (initial body weight [BW]: 257 ± 20.7 kg) were purchased in Texas and Nebraska and transported to the Kansas State University Beef Stocker Unit. Four truckloads were received from February 17 to February 21, 2022. Steers were blocked by truckload (*n*= 4), stratified by body weight, and assigned to pens within each block. Two blocks were stratified across 12 pens (7 to 9 steers per pen) and two blocks were stratified across 8 pens (9 to 11 steers per pen). Within block, pens were randomly assigned to 1 of 4 treatments, resulting in 10 pens per treatment for 40 pens.

Experimental diets are presented in Table 2. The control diet (CON) included (dry matter basis) 39.5% dry-rolled corn, 40% wet-corn gluten feed, 7.5% supplement, and 13% prairie hay. Prairie hay (Table 1) was obtained from central Kansas and contained native warm-season grasses. Non-ground almond hulls and shells replaced prairie hay and were fed at 13% of diet DM (13AH) or replaced prairie hay (13%) and dry-rolled corn (13%) and were fed at 26% of diet DM (26AH). In addition, a subset of almond hulls and shells were ground and replaced prairie hay and were fed at 13% of diet DM (13GAH). For the almond hull and shell processing and digestibility experiments, almond hulls and shells used for 13GAH were ground with a laboratory-scale 1.5 HP Bliss Hammermill (Model 6K630B) using no screen.

**Table 2.** Composition of experimental diets

	Diet^1^			
Item	Control	13AH	13GAH	26AH
Ingredient, % DM				
Dry-rolled corn	39.5	39.5	39.5	26.5
Supplement^2^	7.5	7.5	7.5	7.5
Sweet Bran^3^	40.0	40.0	40.0	40.0
Prairie hay	13.0	—	—	—
Almond hulls	—	13.0	—	26.0
Ground almond hulls	—	—	13.0	—
*Experiment 2*				
Composition, % DM				
Dry matter	75.9	75.1	75.2	74.7
Crude protein	14.2	14.2	14.2	13.6
Organic matter	94.2	94.4	94.4	94.0
Neutral detergent fiber	28.1	22.5	22.4	24.1
Acid detergent fiber	11.5	8.4	8.1	10.2
*Experiment 3*				
Composition, % DM				
Dry matter	75.0	74.8	75.0	75.0
Crude protein	14.9	14.8	14.8	14.2
Organic matter	94.8	94.8	94.8	94.1
Neutral detergent fiber	26.4	22.8	22.7	26.1
Acid detergent fiber	11.0	9.5	9.7	12.8
Calculated composition^4^, Mcal/kg DM				
NEm	1.95	2.00	2.00	1.89
NEg	1.30	1.35	1.35	1.25

^1^Control: prairie hay fed at 13% of diet DM; 13AH: non-ground almond hulls fed at 13% diet DM; 13GAH: ground almond hulls fed at 13% of diet DM; 26AH: Non-ground almond hulls fed at 26% of diet DM.

^2^Supplement pellet formulated to contain (dry matter basis) 9.2% Ca, 5.26% NaCl, and 338 mg/kg monensin. Supplement ingredients: 71.01% wheat middlings, 22.72% calcium carbonate, 5.26% NaCl, 0.39% soybean oil, 0.18% Rumensin 90 (Elanco; Greedfield, IN), 0.11% zinc sulfate, 0.08% manganese (Mn) sulfate (32% Mn), 0.06% vitamin E premix (500,000 IU/kg), 0.05% copper sulfate, 0.10% selenium premix (0.99% Se), 0.008% ethylenediamine dihydriodide (EDDI) premix (9.2% EDDI), and 0.043% vitamin A (600,000 IU/g).

^3^Sweet Bran (Cargill Corn Milling; Blair, NE).

^4^Net energy of maintenance (NEm) and net energy of gain (NEg) were calculated using NASEM (2016) values of diet ingredients.

Due to the large quantity of ground almond hulls and shells required for the receiving trial, almond hulls and shells in experiment 2 were ground using a grinder mixer (Gehl 100; West Bend, Wisconsin) with no screen. Particle size of almond hulls and shells ground using the grinder mixer were larger than those ground using a hammermill. To more accurately estimate particle size, samples ground using the grinder mixer were placed in a 6-sieve stainless steel sieve stack and sifted for ten minutes using a Ro-Tap machine. Four of the six sieves used were larger (15,850, 9,423, 7,925, and 4,000 µm) than those used in experiment 1. The proportion of each sampling that remained in each sieve after sifting was used to calculate geometric mean diameter and geometric standard deviation.

Upon arrival, steers were weighed using a pen scale (Rice Lake Weighing Systems; Rice Lake, WI), placed in soil-surfaced pens within truckload, and fed the control diet at 2.2% of body weight (DM basis) until March 6. On March 6, steers were individually weighed (Silencer, Moly Manufacturing Inc., Lorraine, KS), and a visual identification ear tag was applied. The following day (day 0), steers were individually weighed, vaccinated for clostridial (Vision 7; Merck Animal Health, Kenilworth, NJ) and viral respiratory (Titanium 5; Elanco Animal Health, Indianapolis, IN) pathogens and treated for external parasites (Clean Up II; Elanco Animal Health). Steers were revaccinated for viral respiratory pathogens (Titanium 5; Elanco Animal Health) on day 14.

Individual BW were measured on days 0, 14, and 56. In addition, pen weights were measured weekly using a pen scale, and daily feed delivery was adjusted to 2.2% of pen BW (DM basis). Steers were fed once daily beginning at 0700 hours using a Roto-Mix feed wagon (Model #414-14B; Roto-Mix, Dodge City, KS) for a 56-d period. Individual feed ingredient samples were collected weekly. A portion of each ingredient sample was dried in a forced-air oven at 105 °C for 48 h to determine diet DM. The remaining sample was frozen at −20 °C. Following the completion of the experiment, feed ingredient samples were composited and sent to a commercial laboratory for chemical and proximate analysis.

### Net energy calculations

Performance data were used to calculate net energy for maintenance and net energy for gain provided by the diet as described by Galyean (2019) using NRC (1996) equations. Initial and final body weights were used in the analysis after applying a 4% shrink.

#### Experiment 3: apparent digestibility and ruminal fermentation characteristics

Eight ruminally cannulated heifers (initial BW= 378 ± 44.0 kg) were arranged in a 4 × 4 replicated Latin square to evaluate the effects of almond hull and shell inclusion on ruminal fermentation characteristics and apparent diet digestibility. Experimental diets were identical to those used in experiment 2. Diets were mixed daily using a Marion Mixer (model 2030; Marion, IA) and offered at 2.2% of BW (DM basis). Animals were fed once daily at 1000 hours. The experiment consisted of four consecutive 15-d periods. Data from one heifer in period four were removed due to injury. Each period included 10 d of diet adaptation, 4 d of fecal collection, and 1 d of ruminal fluid sample collection.

Ten grams of chromic oxide (Cr_2_O_3_) were administered intra-ruminally from days 4 to 14 of each period using a 1.5 oz. gel capsule (Torpac; Fairfield, NJ). Individual fecal samples were collected on days 11 through 14 from the rectum of each animal at 8-h intervals. Collection time advanced 2 h each d so each 2-h interval over 24 h was represented. Following collection, fecal samples were composited for each animal within period. Individual feed ingredient samples were collected on days 10 through 14 of each period and were composited within period.

On day 15, digesta samples were collected from 4 separate locations in the rumen prior to feeding. Following 0-h sampling, 3 g of cobalt-EDTA dissolved in 200 mL of water was dosed via the ruminal cannula. Animals were fed, and ruminal digesta samples were collected again at 2, 4, 6, 8, 12, 18, and 24 h post-feeding. Following each collection, samples were strained through 8 layers of cheesecloth. Strained rumen fluid (1 mL) was pipetted into four 2-mL micro-centrifuge tubes containing 250 µL of m-phosphoric acid. In addition, 15 mL of strained rumen fluid was retained for cobalt analysis to estimate liquid passage rate and ruminal liquid volume. Following collection, ruminal fluid samples were immediately frozen (−20 °C) pending analysis. Ruminal pH was measured prior to feeding and again 2, 4, 6, 8, 12, 18, and 24 h post-feeding using a portable pH meter (Pinpoint; American Marine Inc., Ridgefield, CT).

### Laboratory analysis

Individual feed ingredient and fecal samples were sent to a commercial laboratory (SDK Laboratories; Hutchinson, KS) for analysis of dry matter, organic matter ([OM];100—ash; AOAC International, 1999), crude protein (N × 6.25; AOAC International, 1999), neutral detergent fiber (NDF; Van Soest et al., 1991), acid detergent fiber (ADF; Van Soest et al., 1991), calcium (Bowers and Rains, 1988), phosphorus (AOAC International, 1999; procedure 965.17), potassium (AOAC International, 1999; procedure 956.01), and magnesium (AOAC International, 1999; procedure 956.01). Almond hulls and shells were also analyzed for crude fiber as described by AOAC 962.09. Samples collected for ruminal volatile fatty acid (VFA) and ruminal ammonia analyses were centrifuged for 30 min at 17,000 × *g* at 4 °C. Volatile fatty acid concentrations of the supernatant were analyzed using gas-liquid chromatography as described by Vanzant and Cochran, (1994). Ruminal ammonia concentrations of the supernatant were measured as described by Broderick and Kang (1980).

To estimate apparent diet digestibility, approximately 0.5 g of dried fecal material were ground using a 1-mm screen and then placed in a muffle oven at 600 °C for 2 h. Concentrations of chromium within each sample were determined by atomic absorption spectrophotometry as described by Williams et al. (1962). Chromium concentrations in fecal samples were used to estimate total fecal output and diet digestibility according to Cochran and Galyean (1994). Ruminal cobalt concentrations were determined by atomic absorption spectrophotometry. Liquid passage rate was calculated using the nonlinear procedure of SAS (SAS 9.4, SAS Inst. Inc, Cary, NC) by regressing the natural logarithm of cobalt concentration from ruminal samples collected at 2, 4, 6, 8, 12, and 18 h after feeding. Liquid passage rate was determined as the additive inverse of the slope of the regression. Ruminal liquid volume was calculated by dividing the amount of cobalt dosed by the concentration of cobalt at the 0-h intercept of the regression.

### Statistical analysis

Experiment 1 was a randomized complete block design with each particle size reduction run serving as the experimental unit. Each treatment was replicated three times in three separate time periods, and data were analyzed using the MIXED procedure in SAS. The model included a fixed effect of treatment and a random effect of time period. In experiment 2, performance data were analyzed as a randomized block design with a fixed effect of treatment and random effect of block using the MIXED procedure in SAS. In experiment 3, intake, apparent digestibility, and ruminal parameters were analyzed as a replicated Latin square using the MIXED procedure in SAS. The model for intake and apparent digestibility included fixed effects for treatment and period and a random effect for animal. Ruminal pH, ruminal ammonia concentration, and ruminal volatile fatty acid concentrations were analyzed as repeated measures. The model contained fixed effects of treatment, period, hour, and treatment × hour and a random effect of animal. Hour served as a repeated measure and the subject was animal × period. The covariance structure was autoregressive and was selected over compound symmetry and spatial power as determined by Akaike’s information criterion and Bayesian information criterion statistics. When differences among means were indicated by a significant F ratio (*P* ≤ 0.050), treatment means were separated using the method of least significant difference. Significance was declared at *P* ≤ 0.05 and tendencies at 0.05 ≤ *P* < 0.10.

## Results and Discussion

### Experiment 1: almond hull processing

When processed through a hammermill, geometric mean particle size of almond hulls and shells was greatest (*P* < 0.01; Table 3) when no screen was used, intermediate (*P* < 0.01) when a 19.1-mm screen or a 25.4-mm screen was used, and least (*P* < 0.01) when an 11.1-mm screen was used. Particle size standard deviation did not differ (*P* = 0.13) among grinding treatments. When evaluated using the Penn State Particle Separator, the proportion of large particles (> 19 mm) was minor and did not differ (*P* = 0.46) among treatments; however, proportions of medium size particles (8 to 19 mm) tended to be greater (*P* = 0.07) when no screen was used to grind almond hulls and shells compared with an 11.1-mm screen or a 19.1-mm screen. Small particles (4 to 8 mm) comprised 76.5% to 84.3% of ground almond hulls and shells and did not differ (*P* = 0.32) between treatments. Conversely, proportions of fine particles (< 4 mm) increased when hammermill screen size was reduced. Proportions of fine particles were greater (*P* ≤ 0.02) when a 11.1-mm screen was used compared with no screen, a 19.1-mm screen, and a 25.4-mm screen.

**Table 3.** Effects of grinding almond hulls and shells with a hammermill on particle size and bulk density

	Hammermill screen hole diameter^1^					
Item	11.1 mm	19.1 mm	25.4 mm	No screen	SEM^2^	*P*-value^3^
Particle size^4^, µm	1,324^c^	1,772^b^	1,777^b^	2,217^a^	71.2	0.01
Standard deviation	2.53	2.47	2.45	2.18	0.138	0.13
Bulk density, kg/m^3^	542^y^	486^z^	484^z^	476^z^	23.3	0.07
Particle separator^5^, %						
Large	0.00	0.08	0.09	0.33	0.208	0.46
Medium	0.44^z^	2.81^z^	5.12^yz^	15.00^y^	2.091	0.07
Small	79.59	84.25	83.68	76.50	4.581	0.32
Fine	19.92^a^	12.55^by^	11.66^b^	7.92^bz^	2.152	0.01

^1^Almond hulls and shells were ground with a laboratory-scale 1.5 HP Bliss Hammermill (Model 6K630B) using a 11.1 mm, 19.1 mm, 25.4 mm, or no screen. For each screen size treatment, approximately 9 kg of almond hulls were ground at three separate time points to provide three replications per treatment.

^2^Mixed-model standard error of the mean (SEM) associated with comparison of treatment main-effect means.

^3^Treatment main effect.

^4^Geometric mean particle size and standard deviation determined as described by ASABE 319.4 methods.

^5^Determined using the Penn State Particle Separator. Large= particles > 19 mm, medium particles 8 to 19 mm, small particles from 4 to 8 mm, and fine particles < 4 mm.

^a,b,c^Within row, means with unlike superscripts differ (*P* ≤ 0.05).

^y,z^Within row, means with unlike superscripts tend to differ (*P* ≤ 0.10).

Bulk density of the initial non-ground almond hulls and shells was 226.2 kg/m^3^. Grinding almond hulls and shells with a 11.1-mm screen, 19.1-mm screen, 25.4-mm screen, or no screen increased bulk density by 140%, 115%, 114%, and 111%, respectively. In addition, bulk density tended (*P* = 0.07) to be greater when a 11.1-mm screen was used to grind almond hulls and shells compared with when a 19.1-mm screen, 25.4-mm screen, or no screen was used. The increase in bulk density observed with grinding almond hulls and shells could potentially reduce transportation costs. A live-bottom trailer with a load capacity of 80.3 m^3^ could transport approximately 18,160 kg of non-ground almond hulls and shells. Conversely, the same trailer could transport at least 22,700 kg of ground almond hulls and shells and reduce transportation costs by approximately 20%.

Overall, comparisons of almond hull and shell particle sizes and bulk densities are lacking in animal nutrition literature. Because grinding almond hulls and shells with no screen minimized the proportion of fine particles but still achieved an increase of over 100% in bulk density, almond hulls and shells ground with no screen were included as one of the four treatments in the following experiments. As previously mentioned, almond hulls and shells for Exp. 2 were ground using a grinder mixer with no screen. Average geometric mean particle size for almond hulls and shells ground using the grinder mixer was 5,061± 2.1 µm and were 2844 µm larger than the almond hulls and shells used in experiment 3.

### Experiment 2: growth performance

Growth performance data are presented in Table 4. Final BW following the 56-d feeding period were greater (*P* < 0.01) for 13AH and 13GAH compared with 26AH and tended to be greater (*P* = 0.10) for 13GAH compared with CON. In addition, final BW tended to be greater (*P* = 0.06) for CON compared with 26AH. Beckett et al. (1992) reported no differences in growth performance or feed efficiency when ground almond hulls replaced portions of alfalfa and oat hay and were fed up to 15% of diet DM. In addition, final BW and ADG were similar in finishing lambs when almond hulls replaced proportions of chopped alfalfa and were fed up to 10% of diet DM (Phillips et al., 2015). In our experiment, replacing portions of dry-rolled corn with almond hulls and shells reduced final BW; in contrast, Scerra et al. (2022) reported no differences in final BW or ADG when almond hulls replaced up to 30% of barley and maize in a commercial concentrate mix fed to lambs during the final 40 d of the finishing period. In their experiment, hay was provided for ad libitum intake and final almond-hull intake represented approximately 18% of total intake.

**Table 4.** Effects of almond hull and shell inclusion on growth performance of limit-fed growing steers

	Diet^1^					
Item,	Control	13AH	13GAH	26AH	SEM^2^	*P*-value^3^
No. of pens	10	10	10	10		
No. of animals	91	91	91	91		
Body weight, kg						
Day 0	260	262	262	259	1.7	0.22
Day 14	270^y^	270^y^	271^y^	266^z^	2.2	0.09
Day 56	326^aby^	330^a^	333^ax^	319^bz^	3.8	< 0.01
ADG, kg/d						
0 to 14	0.72	0.55	0.68	0.46	0.121	0.15
14 to 56	1.35^bcz^	1.44^aby^	1.47^a^	1.27^c^	0.052	< 0.01
0 to 56	1.19^a^	1.22^a^	1.27^a^	1.07^b^	0.083	0.02
DMI, kg/d						
0 to 14	5.68	5.63	5.68	5.61	0.043	0.23
14 to 56	6.64^a^	6.62^a^	6.66^a^	6.46^b^	0.072	0.04
0 to 56	6.30^a^	6.27^a^	6.32^a^	6.15^b^	0.059	0.03
G:F						
0 to 14	0.129	0.101	0.119	0.083	0.0225	0.20
14 to 56	0.205^b^	0.222^a^	0.222^a^	0.199^b^	0.0078	< 0.01
0 to 56	0.191^aby^	0.198^a^	0.202^a^	0.176^bz^	0.0092	0.04
Diet NEm^4^, Mcal/kg DM	1.72^ab^	1.76^a^	1.78^a^	1.66^b^	0.041	0.02
Diet NEg^5^, Mcal/kg DM	1.10^ab^	1.13^a^	1.15^a^	1.04^b^	0.036	0.02

^1^Control: prairie hay fed at 13% of diet DM; 13AH: non-ground almond hulls fed at 13% diet DM; 13GAH: ground almond hulls fed at 13% of diet DM; 26AH: Non-ground almond hulls fed at 26% of diet DM.

^2^Mixed-model standard error of the mean (SEM) associated with comparison of treatment main-effect means.

^3^Treatment main effect.

^4^Net energy for maintenance, calculated as described by Galyean (2019) based on NRC (1996) requirements.

^5^Net energy for gain, calculated as described by Galyean (2019) based on NRC (1996) requirements.

^a,b,c^Within row, means with unlike superscripts differ (*P* ≤ 0.05).

^x,y,z^Within row, means with unlike superscripts tend to differ (*P* ≤ 0.10).

Average daily gains from days 0 to 56 were greater (*P* ≤ 0.05; Table 4) for 13GAH, 13AH, and CON compared with 26AH; however, ADG from days 14 to 56 were greater (*P* = 0.03) for 13GAH compared with CON and tended to be greater (*P* = 0.09) for 13AH compared with CON. Differences in ADG early in the feeding period may have been associated with diet adaptation and gut fill. Prior to the start of the experiment, all calves were fed CON including prairie hay at 13% of diet DM. On average, commercial nonpareil almond hulls contain approximately 33% nonstructural carbohydrates. Specifically, almond hulls sampled from Northern California contained 10.4% glucose, 8.8% fructose, 5.3% sucrose, 4.6% sorbitol, and 2.5% inositiol (Sequeira and Lew, 1970). When beef cattle were transitioned from fiber-based diets to concentrate-based diets, proportions of fibrolytic bacteria decreased while proportions of nonstructural-carbohydrate fermenting bacteria increased (Fernando et al., 2010); therefore, cattle fed almond hulls and shells in our experiment may have required time to adapt to greater concentrations of nonstructural carbohydrates in the diet compared with those not fed almond hulls and shells.

In addition to diet adaptation, numerical differences in ADG early in the feeding period may have been associated with differences in gut fill. Because all cattle were fed the CON diet prior to trial initiation, calves fed 13AH and 13GAH may have had decreasing gut fill early in the feeding period compared with calves fed CON continuously. As a result, ADG during the first 14 d were numerically less for 13AH and 13GAH compared with CON. After the initial 14 d, gut fill within treatment likely reached a new baseline and remained constant for the remainder of the experiment. Changes in ADG from days 14 to 56 likely reflected changes in BW independent of changes in gut fill.

Aguilar et al. (1984) and Williams et al. (2018) observed no differences in dry matter intake (DMI) when almond hulls replaced portions of alfalfa hay or alfalfa cubes in lactating dairy cattle diets. Conversely, Swanson et al. (2021) reported a cubic response where lactating cows consuming almond hulls at 7% of diet DM had greater DMI compared with those consuming almond hulls at 0%, 13%, and 20% of diet DM. Notably, when expressed as a percent of body weight, DMI were similar among treatments. In our experiment, DMI from days 0 to 56 was greater (*P* ≤ 0.05; Table 4) for 13GAH, 13AH, and CON compared with 26AH. Although DMI was less for 26AH, no feed refusals were present the morning following feed delivery, suggesting almond hulls and shells were readily consumed.

Gain-to-feed (G:F) from days 0 to 56 was greater (*P* ≤ 0.02; Table 4) for 13GAH and 13AH compared with 26AH and tended to be greater for CON (*P* = 0.10) compared with 26AH. In addition, G:F from days 14 to 56 was greater (*P* ≤ 0.04) for 13GAH and 13AH compared with CON and 26AH. Rad et al. (2016) reported no differences in G:F when urea-treated almond hulls replaced alfalfa and were fed at 40% of diet DM to finishing lambs. Similarly, Beckett et al. (1992) and Phillips et al. (2015) reported no reductions in G:F when almond hulls replaced proportions of alfalfa or oat hay and were included in finishing beef cattle or finishing lamb diets.

Differences in BW gains (13GAH, 13AH, and CON vs. 26AH) could partially be associated with differences in DMI. Greater ADG in 13GAH, 13AH, and CON early in the feeding period resulted in greater pen weights and ultimately more feed delivered compared with 26AH. The range in intakes among treatments, however, was relatively modest, and differences among treatments in G:F suggest that the small differences in feed intake did not strongly affect ADG. Overall, data from our experiment agrees with previous literature and demonstrates that almond hulls and shells can be utilized as an alternative to prairie hay in limit-fed growing beef cattle diets without negatively influencing weight gains or feed efficiency.

Dietary net energy concentrations estimated from performance data are presented in Table 4. Dietary concentrations of net energy for maintenance (NEm) and net energy for gain (NEg) were greater (*P* ≤ 0.02) for 13GAH and 13AH compared with 26AH; dietary concentrations of NEm and NEg in CON were intermediate to and did not differ (*P ≥ *0.12) from 13GAH, 13AH, or 26AH. Net energy concentrations calculated using performance data were numerically less than those calculated using tabular values provided by NASEM (2016) but followed a similar trend. Possible reasons for the numerical difference may have been driven by incorrect predictions of the composition of weight gain or environmental factors which influenced performance.

#### Experiment 3: apparent digestibility and ruminal fermentation characteristics

Dry matter intake and organic matter intake did not differ (*P ≥ *0.53; Table 5) among treatments; however, NDF intake was greater (*P* < 0.01) for 26AH and CON compared with 13AH and 13GAH. In addition, ADF intake was greatest (*P* < 0.01) for 26AH, intermediate (*P* < 0.01) for CON, and least (*P* < 0.01) for 13AH and 13GAH. Differences in NDF and ADF intakes were associated with the composition of each diet. Neutral detergent fiber concentrations were 26.1%, 26.4%, 22.8%, and 22.7% of diet DM, while ADF concentrations were 12.8%, 11.0%, 9.5%, and 9.7% of diet DM for 26AH, CON, 13AH, and 13GAH, respectively. Apparent total-tract DM, OM, NDF, or ADF digestibilities did not differ (*P* ≥ 0.15; Table 5) among treatments. Similarly, Can et al. (2007) and Yalchi (2011) reported no differences in DM or OM digestibility when almond hulls replaced portions of alfalfa or wheat straw and were fed to male goats or mature sheep.

**Table 5.** Effect of almond hull and shell inclusion on intake, apparent digestibility, and ruminal fermentation characteristics in limit-fed ruminally cannulated beef heifers

	Diet^1^					
Item,	Control	13AH	13GAH	26AH	SEM^2^	*P*-value^3^
Number of observations	8	7	8	8		
Intake, kg/d						
Dry matter	8.88	8.83	8.87	8.84	0.104	0.61
Organic matter	8.42	8.37	8.29	8.32	0.097	0.53
Neutral detergent fiber	2.34^a^	2.01^b^	1.98^b^	2.30^a^	0.032	< 0.01
Acid detergent fiber	0.97^b^	0.85^c^	0.85^c^	1.14^a^	0.024	< 0.01
Apparent total-tract digestibility, %						
Dry matter	70.1	72.4	73.3	67.8	2.79	0.21
Organic matter	72.8	74.6	75.5	69.8	2.61	0.15
Neutral detergent fiber	54.7	51.4	51.2	46.1	4.61	0.31
Acid detergent fiber	38.9	30.0	33.3	24.1	6.77	0.22
Volatile fatty acids^4^, m*M*						
Acetate	50.2	49.3	49.1	46.5	1.80	0.16
Propionate	24.2^c^	28.4^b^	32.8^a^	24.0^c^	1.66	< 0.01
Butyrate	12.7	13.2	11.4	12.5	0.92	0.22
Valerate	1.8^b^	2.7^a^	3.0^a^	3.0^a^	0.32	< 0.01
Isobutyrate	0.8^a^	0.7^b^	0.7^b^	0.6^c^	0.05	< 0.01
Isovalerate	1.7^y^	1.3^z^	1.5^yz^	1.6^yz^	0.24	0.08
Acetate:Propionate	2.2^a^	1.9^b^	1.7^c^	2.2^a^	0.11	< 0.01
Total volatile fatty acids	91.5^ab^	95.8^ab^	98.4^a^	87.7^b^	3.80	< 0.01
Ruminal ammonia^4^, m*M*	6.0^a^	5.1^ab^	4.4^bc^	3.8^c^	0.85	0.01
Liquid passage rate^5^, %/h	6.3	5.1	5.8	6.2	0.61	0.26
Ruminal liquid volume^5^, L	50.6	52.2	47.8	53.9	2.75	0.16
Ruminal pH	5.96^ab^	5.84^bc^	5.83^c^	5.99^a^	0.065	0.03

^1^Control: prairie hay fed at 13% of diet DM; 13AH: non-ground almond hulls fed at 13% diet DM; 13GAH: ground almond hulls fed at 13% of diet DM; 26AH: Non-ground almond hulls fed at 26% of diet DM.

^2^Mixed-model standard error of the mean (SEM) associated with comparison of treatment main-effect means.

^3^Treatment main effect.

^4^Average of values collected at 0, 2, 4, 6, 8, 12, 18, and 24 h after feeding.

^5^Calculated from samples collected at 2, 4, 6, 8, 12, and 18 h after feeding.

^a,b,c^Within row, means with unlike superscripts differ (*P* ≤ 0.05).

^y,z^Within row, means with unlike superscripts tend to differ (*P* ≤ 0.10).

**Figure 1.**
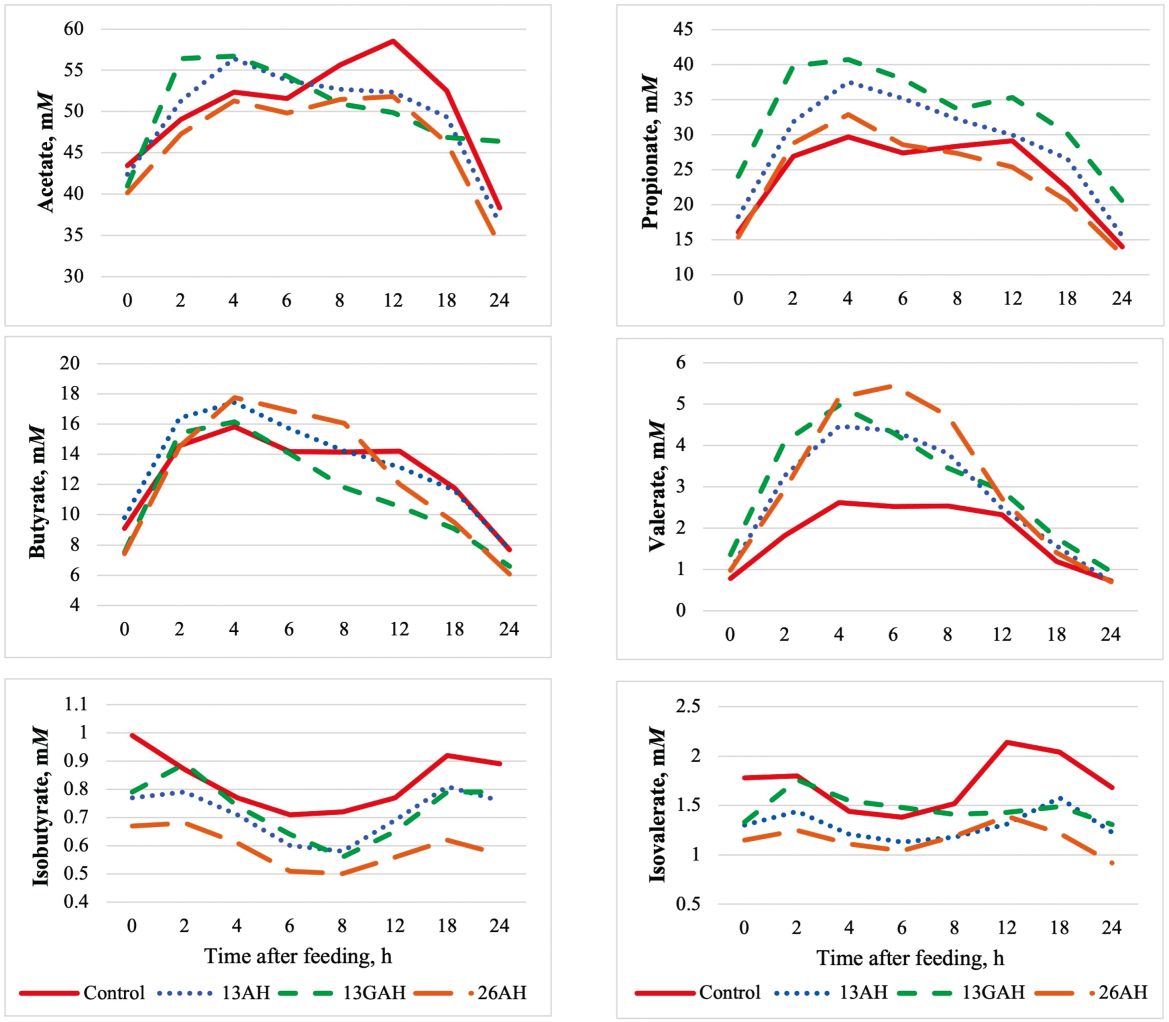
Effects of almond hull and shell inclusion on ruminal volatile fatty acid concentrations in limit-fed growing beef cattle. Control: prairie hay fed at 13% of diet DM; 13AH: non-ground almond hulls and shells fed at 13% diet DM; 13GAH: ground almond hulls and shells fed at 13% of diet DM; 26AH: Non-ground almond hulls and shells fed at 26% of diet DM. Acetate: diet (*P* = 0.16), diet × hour (*P* = 0.54), hour (*P*< 0.01), period (*P* = 0.08), SEM = 3.51. Propionate: diet (*P* < 0.01), diet × hour (*P* = 0.85), hour (*P* < 0.01), period (*P* = 0.08), SEM = 3.32. Butyrate: diet (*P* = 0.22), diet × hour (*P* = 0.37), hour (*P* < 0.01), period (*P* = 0.20), SEM = 1.47. Valerate: diet (*P* < 0.01), diet × hour (*P* < 0.01), hour (*P* < 0.01), period (*P* = 0.20), SEM = 0.47. Isobutyrate: diet (*P*< 0.01), diet × hour (*P* = 0.39), hour (*P* < 0.01), period (*P* = 0.53), SEM = 0.06. Isovalerate: diet (*P* = 0.08), diet × hour (*P* = 0.05), hour (*P* < 0.01), period (*P* = 0.76), SEM = 0.22.

Ruminal VFA concentrations are presented in Table 5, and VFA concentrations over the 24-h sampling period are presented in Fig. 1. Total VFA concentrations were greater (*P* ≤ 0.03) for calves fed 13GAH and 13AH compared with calves fed 26AH. In addition, total VFA concentrations tended (*P* = 0.06) to be greater for calves fed 13GAH compared with calves fed CON. No treatment × hour interactions were observed (*P* ≥ 0.37) for ruminal concentrations of acetate, propionate, butyrate, or isobutyrate. In addition, concentrations of acetate and butyrate did not differ (*P* ≥ 0.16) among treatments; however, propionate concentrations were greatest (*P* < 0.01) for 13GAH, intermediate (*P* ≤ 0.01) for 13AH, and least (*P* ≤ 0.01) for CON and 26AH. As a result, the acetate-to-propionate ratio was least (*P* ≤ 0.03) in 13GAH, intermediate (*P* ≤ 0.03) in 13AH, and greatest (*P* < 0.01) in CON and 26AH. Greater concentrations of propionate in 13GAH and 13AH were likely associated with increased fermentation of non-structural carbohydrates from almond hulls and shells compared with prairie hay.

Ruminal valerate concentrations were greater (treatment × hour: *P* ≤ 0.02) from hours 2 to 6 post-feeding in 13AH, 13GAH, and 26AH compared with CON. As a result, overall concentrations of ruminal valerate were greater (diet effect: *P* < 0.01) in diets containing almond hulls and shells compared with prairie hay. Rad et al. (2016) and Williams et al. (2018) observed no effect of almond hull inclusion on concentrations of ruminal valerate; however, in those experiments almond hulls replaced proportions of alfalfa cubes or alfalfa hay rather than prairie hay. Ruminal concentrations of isobutyrate and isovalerate were minor, but differences among treatments were observed. Concentrations of isobutyrate were greatest (*P* ≤ 0.01) in CON compared with 13AH, 13GAH, and 26AH, whereas concentrations of isovalerate tended (*P* = 0.08) to be greater in CON compared with 13AH. Ruminal concentrations of branched-chain fatty acids increase as dietary protein increases (Dijkstra, 1994); therefore, numerically greater crude protein concentrations in CON may have contributed to increased concentrations of isobutyrate and isovalerate. In addition, greater non-structural carbohydrate fermentation in diets containing almond hulls and shells may have led to greater uptake of isobutyrate and isovalerate by ruminal bacteria compared with diets containing prairie hay.

Ruminal ammonia concentrations were greater (*P* ≤ 0.02; Table 5) in CON compared with 13GAH and 26AH. In addition, ruminal ammonia concentrations were also greater (*P* = 0.05) in 13AH compared with 26AH but did not differ (*P* = 0.37) between 13GAH and 26AH. Differences in concentrations of ruminal ammonia may have been associated with dietary crude protein concentrations. Average crude protein concentrations were 4.3%, 5.0%, and 8.8% of DM for almond hulls and shells, prairie hay, and dry-rolled corn, respectively. Diets were not formulated to be iso-nitrogenous which resulted in lower dietary crude protein when almond hulls and shells replaced prairie hay and proportions of dry-rolled corn. Similar reductions in ruminal ammonia concentrations were observed in lactating dairy cows when almond hulls replaced proportions of alfalfa cubes (Williams et al., 2018). Schwab et al. (2005) indicated that 5 mM of ruminal NH_3_-N was needed to support ruminal microbial growth; therefore, additional protein supplementation may be needed when replacing traditional feed ingredients with almond hulls and shells.

Liquid passage rate and ruminal liquid volume did not differ (*P* ≤ 0.16; Table 5) among treatments; however, ruminal pH was greater (*P* ≤ 0.04; Table 5) in 26AH and CON compared with 13GAH. In addition, ruminal pH was greater (*P* = 0.03) in 26AH and tended (*P* = 0.06) to be greater in CON compared with 13AH. Reduced ruminal pH may have been a result of increased organic matter fermentation and VFA production in 13GAH and 13AH compared with CON and 26AH. Conversely, replacing proportions of dry-rolled corn with almond hulls and shells decreased dietary starch which resulted in a similar ruminal (*P* = 0.72) pH between CON and 26AH. In addition, differences in particle size between almond hulls and shells and prairie hay may have also influenced ruminal pH. Weiss et al. (2017) reported an increase in rumination time and ruminal pH with greater corn stalk particle size. Rumination stimulates saliva production which subsequently increases ruminal pH (Allen, 1997). Time spent ruminating may have been greater in calves consuming CON and 26AH compared with 13GAH and 13AH, which could have contributed to differences in ruminal pH among treatments. Regardless of diet, ruminal pH was within the normal range for beef cattle fed grain-based diets (Nagaraja and Titgemeyer, 2007).

## Conclusions

Replacing prairie hay with almond hulls and shells at 13% of diet DM resulted in similar final BW and ADG following a 56-d feeding period. Greater ADG between days 14 to 56 when almond hulls and shells replaced prairie hay might indicate that cattle may require time to adapt to almond hulls and shells in the diet or that this dietary substitution alters gut fill. Conversely, replacing prairie hay and portions of dry-rolled corn with almond hulls and shells reduced growth performance, ruminal VFA concentrations, and ruminal ammonia concentrations. Grinding almond hulls and shells increased bulk density and numerically improved diet digestibility and performance; however, the value of grinding almond hulls and shells may vary based on location. If almond hulls and shells are transported long distances, grinding prior to shipping could potentially increase the weight of almond hulls and shells transported per load. In addition, a smaller trailer could be used to transport the same weight of ground almond hulls and shells compared with non-ground almond hulls and shells. Overall, our data demonstrate that ground or non-ground almond hulls and shells can be used as an alternative to prairie hay in limit-fed growing beef cattle diets.
